# The effect of a monoclonal antibody to calcitonin-gene related peptide (CGRP) on injury-induced ectopic discharge following lingual nerve injury

**DOI:** 10.1016/j.neulet.2011.09.072

**Published:** 2011-11-14

**Authors:** Katie E. Bowler, Matthew A. Worsley, Lisa Broad, Emmanuel Sher, Robert Benschop, Kirk Johnson, Fiona M. Boissonade, Peter P. Robinson, Julian M. Yates

**Affiliations:** a Unit of Oral & Maxillofacial Medicine & Surgery, School of Clinical Dentistry, University of Sheffield, Claremont Crescent, Sheffield S10 2TA, UK; b Eli Lilly, Erl Wood Manor, Windlesham, Surrey GU20 6PH, UK; c Eli Lilly, Indianapolis, IN 46225, USA

**Keywords:** Lingual nerve injury, Ectopic activity, Calcitonin gene-related peptide, Immunoneutralisation, Monoclonal antibody, Neuropathic pain

## Abstract

The development of ectopic neural discharge at a site of peripheral nerve injury is thought to contribute to the initiation of sensory disturbances and pain. We have previously shown that this discharge can be initiated or increased by the neuropeptide calcitonin gene-related peptide (CGRP). We have now studied a potential therapeutic approach to reducing the discharge by evaluating the effect of a systemically administered monoclonal antibody to CGRP on injury-induced activity in the lingual nerve. In 16 anaesthetised adult ferrets the left lingual nerve was sectioned. One day after the injury, the animals received a subcutaneous injection of either a monoclonal antibody to CGRP or a vehicle control. Three days after the injury, under a second anaesthetic, single-unit electrophysiological recordings were made from central to the injury site (469 and 391 units were analysed in antibody and vehicle groups, respectively), and the proportion of units that were spontaneously active was determined. In the vehicle-treated animals 6.4 ± 2.7 [SEM]% of the units were spontaneously active, with conduction velocities of 8.8–40.8 m/s and discharge frequencies of 0.03–2.7 Hz. In the monoclonal antibody-treated animals 5.7 ± 2.0% of the units were spontaneously active, with conduction velocities of 13.9–38.8 m/s and discharge frequencies of 0.07–1.8 Hz. There was no significant difference between these two groups (for spontaneous activity and conduction velocity: *p* > 0.05, Student's *t*-test; for discharge frequency: *p* > 0.05, Mann–Whitney test), suggesting that the spontaneous activity initiated by a nerve injury cannot be modulated by administration of a monoclonal antibody to CGRP.

## Highlights

► Previously we showed that CGRP can increase ectopic discharge at nerve injury sites. ► Here we monitor the effect of anti-CGRP antibody on injured nerve fibre activity. ► Systemic anti-CGRP does not modify activity in injured lingual nerve fibres. ► Activity in injured lingual nerve fibres is not affected by sequestration of CGRP. ► Other modulators may offer better therapeutic targets for nerve injury-induced pain.

## 1 Introduction

Shortly after sectioning a peripheral nerve, the damaged axons start to behave abnormally [8,14]. Some axons discharge action potentials spontaneously in the absence of any stimulus, and others respond to gentle mechanical distortion of the injury site. The discharge is thought to result from alterations in the expression of ion channels and other regulators of neuronal excitability within the damaged axons. This centrally directed ectopic activity is thought to contribute to the pain and dysaesthesia experienced by some patients, and reduction of the discharge may provide the basis for future pharmacological treatment [7].

We have previously studied injury-induced ectopic activity in the lingual nerve, a branch of the trigeminal nerve that is susceptible to iatrogenic damage during routine surgical procedures, such as the removal of lower third molars [12]. We showed that 3 days after sectioning the nerve in anaesthetised adult ferrets, up to 36% of the axons became spontaneously active and up to 35% were sensitive to mechanical stimulation [15]. In parallel immunocytochemical studies, we found an accumulation of neuropeptides at the injury site, and the maximum accumulation of peptides coincided with the periods of greatest spontaneous activity [2]. One of the neuropeptides present was calcitonin gene-related peptide (CGRP) and, in view of its known role in neural transmission and neuromodulation [13], we hypothesised that it might modify the abnormal discharge after nerve injury. This possibility was confirmed in studies on another branch of the trigeminal nerve, the inferior alveolar nerve, in which topical or close-arterial application of CGRP or a CGRP antagonist was found to initiate or modulate the discharge from some damaged axons [9]. Here we have pursued a novel approach to modifying the action of CGRP on damaged axons, using systemic administration of a monoclonal antibody to CGRP two days prior to electrophysiological recordings; we have also reverted to the lingual nerve as our experimental model.

## 2 Methods

Sixteen adult female ferrets aged 5–8 months and weighing 0.7–1.1 kg were used in this investigation, and all procedures were undertaken in accordance with the UK Animals (Scientific Procedures) Act, 1986. Under anaesthesia (ketamine, 25 mg/kg; xylazine, 2 mg/kg; i.m.), an incision was made in the left submandibular region and the mylohyoid muscle split to expose the left lingual nerve lying on the pharyngeal constrictor muscle. The nerve was sectioned using micro-scissors and left in alignment. The incision was closed and a single dose of antibiotic was administered (ampicillin 22.5 mg/kg, i.m.; Duphacillin, Fort Dodge, UK). One day later, the animals received a subcutaneous injection of either a monoclonal antibody to CGRP (Sigma–Aldrich, USA; 2 mg/ml administered at 1 ml/kg, 8 animals) or the phosphate-buffered saline (PBS) vehicle (8 animals). The antibody had previously been dialysed in PBS using Slide-A-Lyzer dialysis cassettes (Pierce, Rockford, IL, USA) to remove the 15 mM sodium azide.

**Fig. 1:**
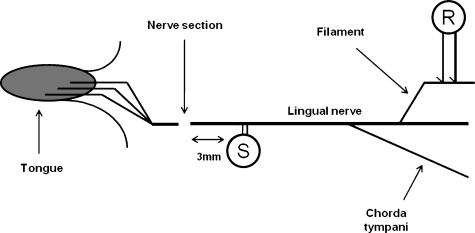
A diagrammatic representation of the recording arrangement. ‘S’ denotes the site of stimulation and ‘R’ denotes the site of the recordings, central to both the injury site and the junction with the chorda tympani.

On the third day post-injury, the animals were re-anaesthetised with sodium pentobarbitone (induction 40 mg/kg i.p.; maintenance 2.5–10 mg/kg i.v. as required), the trachea was cannulated, the ECG recorded, and body temperature was maintained at 38 ± 0.5 °C with a thermostatic heating blanket. The animal was prepared for the electrophysiological recordings as previously described [15], and the investigator was kept blind to the experimental group. In brief, the lingual nerve was exposed at the injury site and central to its junction with the chorda tympani, by removal of the ramus and part of the body of the mandible. It was then covered in warm liquid paraffin in a pool created from the surrounding skin. After dividing the epineurium with a razor chip, fine filaments were dissected from the nerve and placed directly on platinum wire electrodes (0.15 mm diameter). To avoid centrally mediated spontaneous discharge, the nerve was sectioned centrally. A pair of stimulating electrodes was placed across the nerve approximately 3 mm central to the injury site, and isolated from the underlying muscle with a small piece of parafilm (Parafilm ‘M’, American National Can, USA). The experimental and recording arrangements are illustrated diagrammatically in Fig. 1.

For each filament, the following characteristics were determined:

(a) *The number of units in the filament*
  This was determined on an oscilloscope by assessing recruitment of individual units in response to steadily increasing electrical stimuli of 0.1 ms duration and up to 10 V applied to the electrodes near the injury site.
(b) *The number of units that were spontaneously active*
  After initial assessment on the oscilloscope, any activity was recorded on disk and analysed using a 1401 Plus interface and Spike 2 for Windows software (Cambridge Electronic Design Ltd., Cambridge, UK). This software allows spontaneously active units to be identified based on their action potential shape and amplitude. The discharge frequency of each unit was determined and categorised as regular or irregular. The proportion of spontaneously active units in antibody- and vehicle-treated groups was compared using an unpaired Student's *t*-test. The discharge frequency of spontaneously active units in the two groups was compared using an unpaired Mann–Whitney test. In both cases significance levels were set at *p* < 0.05.
(c) *The conduction velocity of each unit*
  This was calculated for each unit from the latency of the response to the electrical stimuli applied at the injury site. The conduction velocity of spontaneously active units in the two groups was compared using Student's *t*-test.

To assay antibody levels, approximately 1 ml of blood was collected from a superficial vein in the hind limb at the end of each experiment, placed into EDTA-coated tubes, centrifuged at 4 °C for 5 min at 5000 rpm, and the plasma stored at −80 °C. Analysis to quantify antibody levels was undertaken using an enzyme-linked immunosorbant assay (ELISA). ELISA plates were coated overnight (4 °C) with anti-mouse IgG1 (BD Biosciences, San Jose, USA) in carbonate coating buffer (pH 9.6), washed three times with wash buffer (0.02 M Tris pH 7.4, 0.15 M NaCl, 0.1% Tween-20) and blocked for 1 h at room temperature with 1% bovine serum albumin (BSA) in wash buffer. Plates were washed again prior to the addition of plasma samples in duplicate. The samples were tested at 100, 400, 1600 and 6400 times dilutions. The captured antibody was detected using an anti-mouse kappa-HRP (BD Biosciences, San Jose, USA) and the assay was developed using o-phenylenediamine (Sigma–Aldrich, St. Louis, USA) for 6 min and the reaction was stopped by adding 1 N HCl. A standard curve of the purified anti-CGRP antibody was used to calculate the plasma concentration.

## 3 Results

**Fig. 2:**
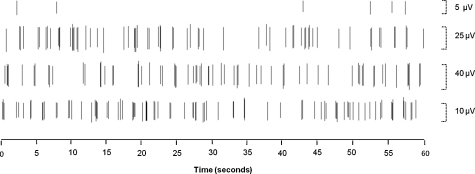
Spontaneous activity (SA) recorded in a unit dissected from the lingual nerve 3 days after injury. Action potentials have been identified using the spike discrimination software, and four spontaneously active units are shown.

**Fig. 3:**
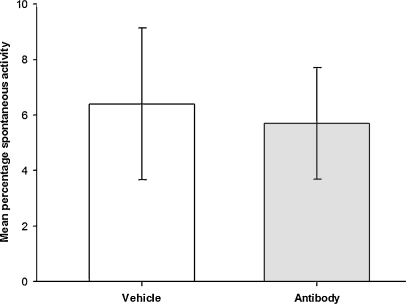
Mean (±SEM) percentage of units in the lingual nerve that were spontaneously active 3 days post injury.

No apparent adverse reactions were observed during the monitoring period post-vehicle or antibody administration. A total of 469 units in 145 filaments were analysed electrophysiologically in the antibody group (mean 58 units per animal, range 33–95) and a total of 391 units in 143 filaments were analysed in the vehicle group (mean 48 units per animal, range 18–83). Spontaneously active units were recorded in both treatment groups and examples can be seen in Fig. 2. Fig. 3 shows the mean percentage of spontaneously active units in vehicle and antibody treated animals. In the antibody group a total of 28 units were spontaneously active (range 0–9 units per animal) whilst in the vehicle group, 21 spontaneously active units were recorded (range 0–10 units per animal). There was no significant difference between the two groups in terms of the proportions of spontaneously active units obtained from each animal: the mean proportion of units with spontaneous activity was 5.7% ± 2.0 [SEM] in the antibody group, and 6.4% ± 2.7 in the vehicle group (*p* = 0.82, unpaired Student's *t*-test; Fig. 3).

**Fig. 4:**
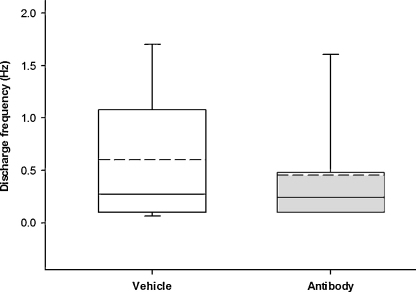
Box and whisker plots showing the mean and median discharge frequencies recorded in spontaneously active units from vehicle and antibody treated animals. The dotted line represents the mean discharge frequency and the solid line represents the median.

All of the spontaneously active units recorded in both groups had an irregular discharge pattern (interspike interval deviation > 50 ms). There was no significant difference in discharge rates between the treatment groups: discharge rates ranged from 0.07 to 1.82 Hz (median 0.24 Hz) for animals in the antibody group, and from 0.03 to 2.7 Hz (median 0.27 Hz) for animals in the vehicle group (*p* > 0.05, unpaired Mann–Whitney test, Fig. 4).

**Fig. 5:**
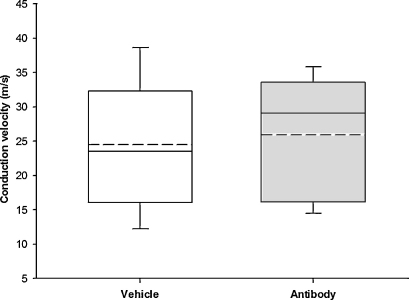
Box and whisker plots showing the mean and median conduction velocity recorded for the spontaneously active units found in each treatment group. The dotted line represents the mean conduction velocity and the solid line represents the median.

There was no significant difference in conduction velocities of the spontaneously active units between the two groups. Conduction velocity ranged from 13.9 to 38.7 m/s (mean 25.9 ± 2.0 m/s) in the antibody-treated animals and from 8.8 to 40.8 m/s (mean 24.5 ± 2.05 m/s) for the vehicle-treated animals (*p* = 0.63, unpaired *t*-test, Fig. 5).

The CGRP antibody assays revealed average plasma concentrations ranging from 4.82 to 9.02 μg/ml (mean 6.31 ± 0.76 μg/ml) in the treated animals. No antibody was detected in animals that had received the vehicle.

## 4 Discussion

In common with our previous studies on the response of damaged trigeminal nerves to injury [4,15], we found spontaneously discharging units in most animals three days after nerve section. All of these units had irregular discharge patterns, low discharge frequencies and included myelinated fibres across a broad range of conduction velocities. In the present experiments, we have shown that systemic administration of a monoclonal antibody to CGRP does not reduce the proportion of lingual nerve axons that develop an ectopic discharge following injury, nor does it change the discharge frequency. Furthermore, the range of conduction velocities of the spontaneously discharging axons was unaltered, suggesting that there was no change in the fibre population that was affected.

In the present study, we were not able to assess levels of spontaneous activity in all fibre types as we were not able to discriminate single unmyelinated fibres. Also, for technical reasons, it was not possible to establish the effects of the antibody on mechanical sensitivity. Thus, it is possible that the antibody may have had some effect on these parameters.

The use of a systemically administered antibody for pain modulation is a novel approach and we have previously shown that the same antibody reduces central neuronal activation in a trigeminal inflammatory pain model [5,6]. In those studies, Fos expression in the trigeminal nucleus was quantified in response to stimulation of the inflamed ferret tooth pulp, and was significantly lower after antibody administration. The dose and time course used, and plasma concentrations achieved were very similar to those of the present investigation. This dose of antibody has also been shown by Eli Lilly to inhibit dural plasma extravasation induced by stimulation of the trigeminal ganglion (http://www.wipo.int/patentscope/search/en/WO2007076336). Thus, it seems likely that the plasma concentrations in the present study would have been sufficient to sequester CGRP at the injury site. Taken together these data suggest that this approach may be effective for inflammatory but not neuropathic pain. Further studies utilising appropriate behavioural models are necessary to establish this.

This result was surprising as evidence for a potential role for CGRP in the aetiology of injury-induced spontaneous discharge is compelling. In addition to the known accumulation of CGRP in the damaged axons [2,3] and effects of CGRP and CGRP antagonists when applied locally [9], there is also clinical evidence that damaged lingual nerves have higher levels of CGRP in patients suffering from pain than in patients without pain [11]. The mechanism by which the CGRP might exert an influence is not clear and it could act indirectly rather than directly on the neuronal tissues. One such mechanism could be via the vasculature as CGRP is a potent vasodilator, and Zochodne et al. [17] have demonstrated a local reduction in blood flow when CGRP antagonist was applied topically to a sciatic nerve neuroma. Another possibility is that CGRP could affect the neuronal discharge through modulation of immunological and inflammatory responses [10], which are also thought to play a role in neuropathic pain. However, the data from the current study indicate that blocking the effect of CGRP alone does not affect the level of spontaneous discharge.

Alternative methods for modification of spontaneous discharge from injured axons must therefore be sought. Carbamazepine, a membrane stabilising drug, and SB-750364, a TRPV1 receptor antagonist, have both been shown to reduce spontaneous discharge from some damaged lingual nerve axons, whilst others are not affected [1,16]. Such drugs might therefore be expected to give partial relief of symptoms, and use of carbamazepine in patients with dysaesthesia confirms this effect in some individuals [12]. More universally effective treatments are required.

## Contributors

Bowler carried out majority of laboratory work and analysis, and wrote initial draft of manuscript. Yates: laboratory supervision of electrophysiology, and contribution to manuscript. Worsley: project supervision. Robinson, Boissonade: initiation and supervision of project, and preparation of final manuscript. Johnson advised on use and dialysis of antibody. Benschop: analysis of blood samples. Broad, Sher: industrial supervisors of project.

## Role of the funding source

The BBSRC together with Eli Lilly funded a doctoral training grant to FMB, PPR and JMY. Eli Lilly carried out the analysis of the blood samples, and has approved the submission of the manuscript.
